# A Rare Case of Diverticulitis With a Colo-Adnexal Fistula

**DOI:** 10.7759/cureus.47017

**Published:** 2023-10-14

**Authors:** Varsha Gopalan, William G Baker, Shayla Albright, Benjamin Crawshaw M.D.

**Affiliations:** 1 Obstetrics and Gynecology, American University of Antigua, New York, USA; 2 Surgery, American University of Antigua, New York, USA; 3 Pediatrics, American University of Antigua, New York, USA; 4 Colorectal Surgery, MetroHealth Medical Center, Cleveland, USA

**Keywords:** hartmann’s procedure, abcess, diverticulitis, fistula, colo-adnexal

## Abstract

Diverticulitis is a very common cause of hospitalization in the United States with fistula formation being a common complication. However, a fistula formation between the sigmoid colon and an ovary is an exceptional rarity. We present a case of a 22-year-old female with a colo-ovarian fistula misdiagnosed as a tubo-ovarian abscess after a recent episode of diverticulitis. Initial workup, imaging studies, and treatment ending with Hartmann's procedure with eventual colostomy reversal are described. A review of similar cases within the literature and fistulas is also presented.

## Introduction

Diverticular disease can be defined as symptomatic diverticulosis due to diverticulitis or its complications [1]. Diverticulitis is the inflammation or infection of one or many colonic diverticula. The most common risk factors are advancing age, constipation, consumption of red meat, elevated body mass index (BMI), smoking, immunosuppressive agents such as chemotherapy or corticosteroids, NSAIDs, menopausal hormone therapy, and opiate usage. The most common site for diverticulitis is the sigmoid colon. Diverticulitis usually presents with low-grade fevers, left lower quadrant pain, sterile pyuria, a tender palpable mass due to pericolonic inflammation, and changes in bowel habits, such as constipation or diarrhea. Some of the common complications seen in patients with diverticulitis include perforation, abscess formation, intestinal obstruction, and fistulas [2]. We present a case with a primary focus on fistula formation after acute diverticulitis.

Fistulas are an abnormal connection between two surfaces caused by local inflammation or iatrogenic injury, and generally fistulas in diverticular diseases present with pneumaturia and fecaluria [2]. According to the literature, the rate of fistula formation is between 17% and 27% with the most common type being a colo-vesical fistula. However, a fistula can form between any two epithelial surfaces. The different types of fistula include colo-vesical, colo-vaginal, colo-salpingeal, colo-enteric, and colo-cutaneous fistulas. Colo-cutaneous fistulas are rare and occur within 1-4% of the total number of fistulas complicating colonic diverticular disease [3]. Colo-enteric fistulas have an occurrence rate of 3% of diverticulitis cases [4]. Colo-vaginal fistulas are the second most common type of fistula formation after diverticulitis, accounting for roughly 25% of fistulas from diverticulitis complications. Colo-salpingeal is one of the rarest fistulas accounting for <1% of diverticulitis fistulas [5].

We present a case discussing a unique scenario - fistula formation between the colon and an adnexal structure following an episode of acute diverticulitis. This occurrence is quite uncommon, with very limited documented cases in the medical literature.

## Case presentation

A 22-year-old female with a past medical history of morbid obesity with a BMI of 48 and recent diverticulitis with a Hinchey classification of 1a, which was medically resolved three weeks prior, presented to the emergency department with severe abdominal pain, nausea, diarrhea, and vomiting for one week. Her white blood cell count was found to be 11,100 mm^3^ with neutrophilic prominence, and a pregnancy test was negative. A physical exam showed an obese habitus, a soft but distended abdomen, and diffuse abdominal tenderness being worse in the left lower quadrant. A pelvic exam was done revealing a milky vaginal discharge with no suprapubic or cervical motion tenderness. The patient was not menstruating at this time. She reported regular cycles of 26-30 days and no sexual activity with no change in the character of menstrual blood. The patient's vital signs showed a blood pressure of 141/99 mmHg, a respiratory rate of 28 breaths/minute, a temperature of 99.6℉, a heart rate of 119 beats/minute, and an oxygen saturation of 99% on room air.

**Figure 1:**
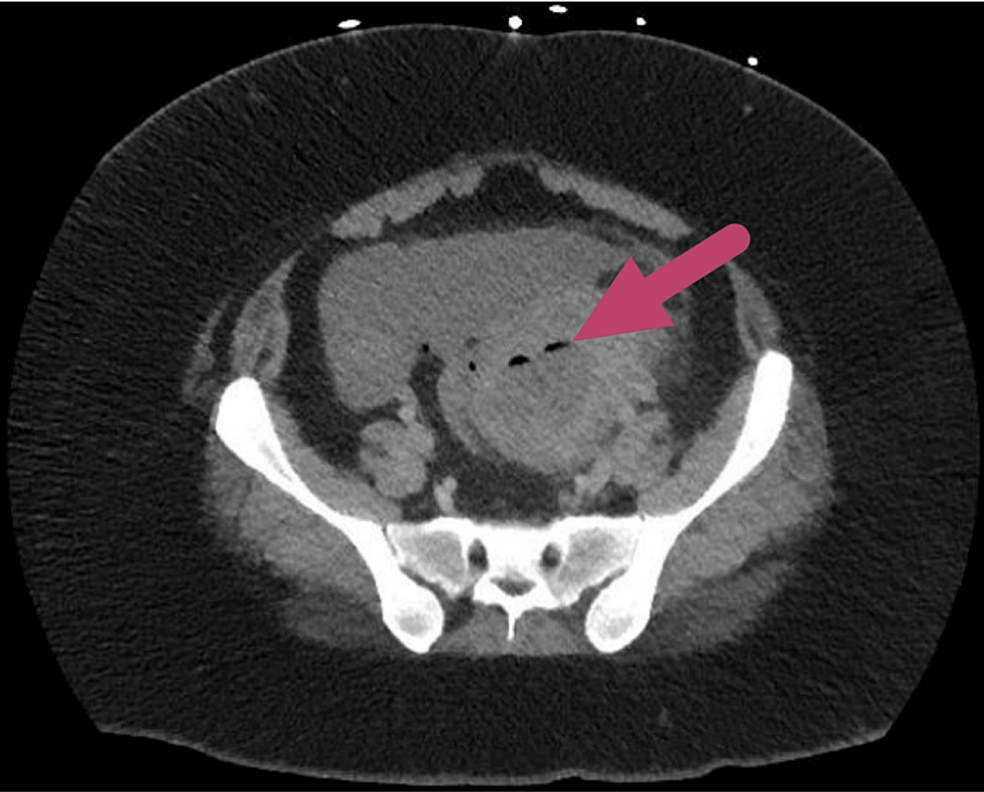
Computed tomography showing the left adnexal mass with internal air (red arrow).

**Figure 2:**
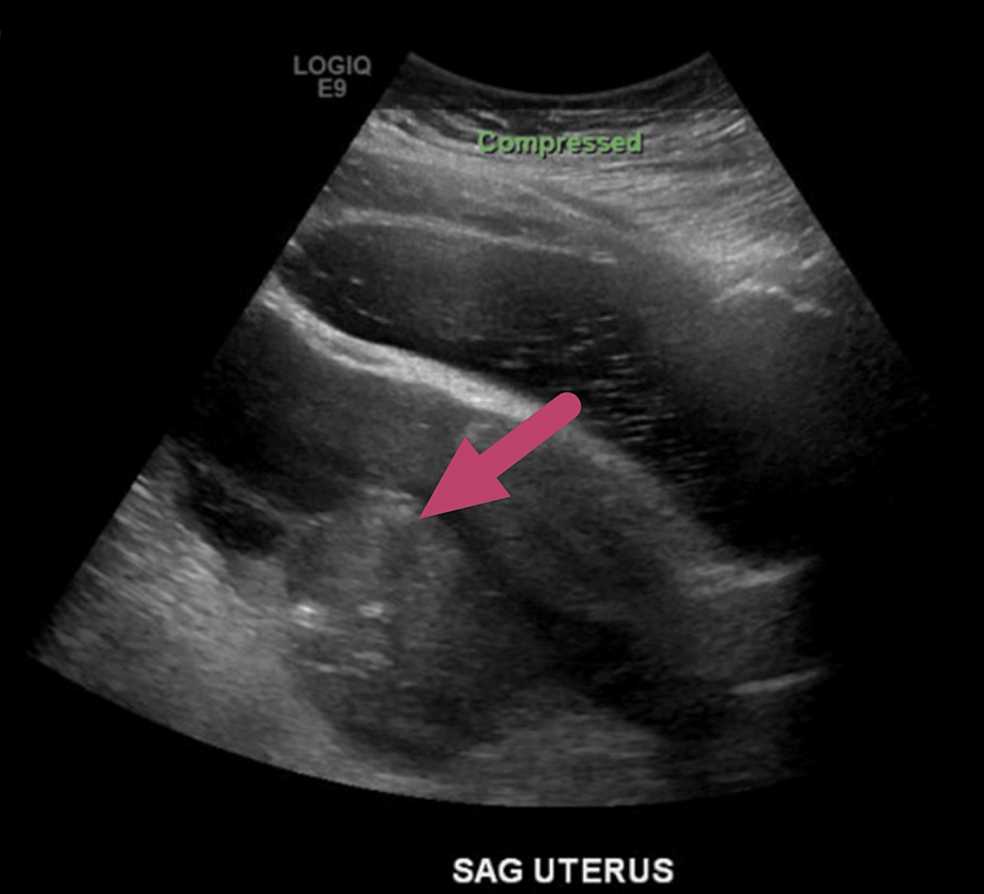
Ultrasound of the female pelvis and bladder showing a left adnexal mass (red arrow).

Radiologic images were collected to better determine the etiology. A contrast abdominal and pelvic computed tomography (CT) showed a left adnexal mass with surrounding inflammatory changes, internal air, and mild ascites (Figure 1). Due to these abnormal results, an ultrasound of the pelvis and bladder was ordered, which showed loculated free fluid present within the pelvis and a 7.4 cm x 4.6 cm x 5.7 cm heterogeneous mass with internal air present in the left adnexa concerning tubo-ovarian abscess (Figure 2). The patient was then admitted and interventional radiology placed a drain into the abscess, but the patient continued to deteriorate. A more recent physical exam revealed rebound tenderness, guarding, rigidity, and her vital signs were significant for a heart rate of 124 beats/minute.

Obstetrics and gynecology was consulted for a suspected tubo-ovarian abscess, and she was taken to the operating room for a diagnostic laparoscopy under general anesthesia in which extensive adhesions and large amounts of green-yellow fluid were found. The decision was made to convert to an exploratory laparotomy, which found four quadrant feculent peritonitis, with approximately 1,000 cc of purulent fluid. Upon further exploration, a 1 cm colotomy was found in the sigmoid colon. A colo-adnexal fistula was found to be the source of the abscess. The pelvis was frozen due to intense inflammatory reactions with extensive adhesions.

A decision was made to proceed with Hartmann's procedure (sigmoidectomy with end colostomy), and two drains were intentionally left in place. The patient tolerated the procedure well without any complications. Gross specimens were then sent to pathology, which revealed an 11 cm x 10 cm x 7 cm abscess with adherent gray exudate, a 2.5 cm hemorrhagic abscess cavity, and multiple mucosal outpouches consistent with diverticula. No masses or lesions were identified. The patient tolerated the procedure well and was discharged home on postoperative day eight. 

Her postoperative course was uncomplicated with no changes in menstruation and normal 26-30 day cycles. She returned nine months later for a colostomy reversal. A laparoscopic-assisted end colostomy reversal was performed under general anesthesia, which showed a large parastomal hernia, minimal adhesions, and a successful end-to-end colorectal anastomosis after rigid proctoscopy showed no air leaks. The patient tolerated the procedure well and was discharged home on postoperative day three.

## Discussion

Our case here represents a colo-adnexal fistula, which is a rare complication of diverticular disease. As mentioned earlier, a colo-vesicular or a colo-vaginal fistula is more common after an acute episode of diverticulitis than a fistula between the colon and ovaries or the colon and fallopian tubes. Most research articles addressing colo-adnexal fistulas occur in patients with ovarian neoplasm, polycystic ovary syndrome, pelvic inflammatory disease, previous surgical procedures, radiation exposure history, or patients with Crohn’s disease [6]. The case reports mentioned below are exclusively colo-adnexal fistulas seen after diverticulitis with two cases discussed in detail.

A case report and literature review published in the International Journal of Surgery Case Reports discussed two cases of colo-ovarian fistula after an episode of diverticulitis [6]. The first case was that of a 29-year-old female with a past medical history of polycystic ovarian syndrome and medically managed recurrent diverticulitis who presented with a colo-ovarian fistula three months after a previous episode of diverticulitis. The patient had persistent left lower quadrant pain and underwent a laparoscopic evaluation for a suspected rare fistula. Upon visualization, the sigmoid colon was seen to be fixed to the left ovary, and the decision was made to resect the sigmoid colon, left ovary, and fallopian tube. Another case was that of a 53-year-old female with a past medical history of acute diverticulitis who presented with abdominal pain and fever 12 days after completing a 10-day course of inpatient intravenous antibiotics for an acute diverticulitis episode. Similar to the patient above, she also had persistent left lower quadrant pain and was referred to laparoscopic evaluation where significant inflammatory reactions were seen in the sigmoid colon, left ovary, and fallopian tube, and subsequent resection of these regions was performed. One of the main differences between the two cases above is the time difference in fistula formation between patients who are acute or chronic. Although the patient in our case was a 22-year-old, a colo-adnexal fistula after diverticulitis can be formed in any age group, as reported by the cases presented in this review.

In our case, the initial clinical symptoms of milky vaginal discharge and radiological findings of an adnexal mass with inflammation, internal air, and free fluid in the pelvis pointed toward a tubo-ovarian abscess. Diagnostic laparoscopy was superior to imaging techniques for our case in diagnosing the fistula secondary to acute diverticulitis due to inconclusive results from computed tomography. Our patient’s clinical course, although different in age group, was similar to the second case discussed in the above literature. Our patient deteriorated despite percutaneous drainage by interventional radiology, and surgery had to be performed sooner with Hartmann's procedure. The first case described above had favorable conditions to delay surgery and perform the resection with a primary anastomosis. Few cases have been described of diverticulitis with colo-adnexal fistulas, which are described below in Table 1.

**Table 1:** Case reports of colo-adnexal fistulas and their age, symptoms, imaging findings, treatment, and outcome for each case.

Article	Age	Symptoms	CT/MRI findings	Treatment	Outcome
Jangam et al. (2022) [7]	65	Left lower quadrant pain, fever, diarrhea, & weight loss	Initial CT showed left adnexal lesion with air-fluid levels and a normal colon. Pelvic ultrasound showed sigmoid thickening. Misdiagnosed as a tubo-ovarian abscess.	Laparoscopic anterior resection & en bloc left salpingo-oophorectomy	Full recovery without any complications
Syllaios et al. (2018) [8]	51	Recurrent UTIs, stool and gas leaking from the vagina	Pelvic MRI only showed diverticular disease and a fistula between the colon and posterior vaginal wall. Laparotomy showed a short fistula between the colon and left ovary.	Low anterior resection of the sigmoid colon, fistulectomy, left salpingo-oophorectomy with end-to-end anastomosis	Full resolution of her presenting symptoms
Rosenzweig et al. (2017) [9]	54	Abdominal pain, bloody vaginal discharge, and recurrent UTIs. Asymptomatic diverticular disease	Transvaginal ultrasound showed a hyperechoic structure in the fundus of the uterus and avascular complex cyst. CT showed multiple areas of gas within the left ovary and a fistula connecting to the sigmoid colon. Misdiagnosed as retained intrauterine device.	En bloc laparoscopic resection of the colon, left ovary, and a small portion of the fallopian tube with end-to-end anastomosis	Full resolution of her presenting symptoms
Darii Plopa et. al (2020) [10]	69	Recurrent vaginal discharge positive for Escherichia coli and Streptococcus constellatus and a history of diverticulitis	CT scan and hysterosalpingography showed the colo-fallopian tube fistula.	Sigmoid resection with concomitant left salpingo-oophorectomy with end-to-end anastomosis	Unremarkable postoperative course with negative cultures at 1 month follow up
Jones et al. (2023) [11]	65	Recurrent malodorous vaginal discharge positive for Escherichia coli and a history of diverticulitis	CT scan failed to show any pathologies. Diagnostic laparoscopy showed a colo-fallopian fistula and a colo-vaginal fistula.	En bloc laparoscopic resection of the colon with end-to-end anastomosis	No recurrent infections with E. coli were noted after the surgery and an unremarkable postoperative course

The many cases mentioned above were initially misdiagnosed as alternative pathologies, emphasizing the complex and variable presentations of colo-adnexal fistulas. Further complicating the diagnosis, conventional imaging techniques failed to definitively identify the presence of such fistulas. Instead, these modalities primarily detected diverticular disease. One recurring observation from the cases above, including our case, that could aid in the diagnosis of a colo-adnexal fistula is the presence of gas within the adnexa. Lastly, due to the rarity of the condition, diagnostic and treatment guidelines do not have a current systematic approach. From our review of the literature, the presence of recurrent vaginal discharge or urinary tract infections and a history of diverticulitis should raise suspicion and warrant further investigation for a possible colo-adnexal fistula. Lastly, with regard to treatment approaches, our review of cases has shown that initial treatment using conservative management or drainage-only techniques was met with failure. The treatment plan with the most favorable outcome in these cases has shown to be a laparoscopic resection of the fistulizing structures with end-to-end anastomosis. Hartmann’s procedure was used in emergency cases, especially in patients with sepsis, and had a favorable prognosis for colostomy reversal, as evidenced in our case as well as the cases mentioned above.

## Conclusions

Diverticulitis, although common in older adults, is a rarity in younger patients with only 10% of known cases being below the age of 40. ​​Fistula formation between adnexal structures and the intestine is an exceptional rarity. Both of the above rarities cause misdiagnosis and delays in the management of these fistulas, especially in the younger population. As more cases of colo-adnexal fistulas arise within the literature, there is hope of finding a definitive guideline and approach to both diagnosis and treatment of these types of fistulas. Our case report could add to the few available cases within the current literature to help diagnose, treat, and minimize usual and unusual complications of colo-adnexal fistulas following diverticulitis.

## References

[1] Pemberton Pemberton, J. H., & Peery, A. (2023, January 5 (2023). Clinical manifestations and diagnosis of acute colonic diverticulitis in adults. https://medilib.ir/uptodate/show/2633.

[2] Pemberton JH (2023). Acute colonic diverticulitis: surgical management. UpToDate.

[3] Bahadursingh AM, Virgo KS, Kaminski DL, Longo WE (2003). Spectrum of disease and outcome of complicated diverticular disease. Am J Surg.

[4] Ahmad DS, Quist EE, Hutchins GF, Bhat I (2016). Coloenteric fistula in a young patient with recurrent diverticulitis: a case report and review of the literature. Neth J Med.

[5] Vilallonga R, Baena JA, Fort JM, Gonzalez O, Gemar E, Armengol Carrasco M (2009). Colouterine fistula complicating diverticulitis in elderly women. Int J Colorectal Dis.

[6] Quintela C, Santos C, Silva AC, Barbosa E, Silva AR, Silva A (2020). Colo-ovarian fistula complicating acute diverticulitis: two cases and literature review. Int J Surg Case Rep.

[7] Jangam A, Gillespie C (2022). A rare case of colo-salpingeal fistula complicating acute sigmoid diverticulitis. J Surg Case Rep.

[8] Syllaios A, Koutras A, Zotos PA (2018). Colovaginal and colo-ovarian fistula at a patient with asymptomatic diverticular disease. J Surg Case Rep.

[9] Rosenzweig M, Marshall J, White RA, Tismenetsky M, Shembde D (2017). Colo-ovarian fistula. J Surg Case Rep.

[10] Darii Plopa N, Gica N, Gerard M, Nollevaux MC, Pavlovic M, Anton E (2020). A very rare case of colosalpingeal fistula secondary to diverticulitis: an overview of development, clinical features and management. Medicina (Kaunas).

[11] Jones DH, Spielmann SM, Falconi S, Obokhare I (2023). Colo-fallopian fistula: a rare complication of sigmoid colon diverticulitis. Cureus.

